# Synthesis of Terpyridine-Terminated Amphiphilic Block Copolymers and Their Self-Assembly into Metallo-Polymer Nanovesicles

**DOI:** 10.3390/ma12040601

**Published:** 2019-02-17

**Authors:** Tatyana Elkin, Stacy M. Copp, Ryan L. Hamblin, Jennifer S. Martinez, Gabriel A. Montaño, Reginaldo C. Rocha

**Affiliations:** 1 Los Alamos National Laboratory, Center for Integrated Nanotechnologies, Los Alamos, NM 87545, USA; tatyana_elkin@lanl.gov (T.E.); smcopp@lanl.gov (S.M.C.); rhamblin@lanl.gov (R.L.H.); jennifer.martinez@nau.edu (J.S.M.); 2 Center for Materials Interfaces in Research and Applications and Program of Applied Physics and Materials Science, Northern Arizona University, Flagstaff, AZ 86011, USA

**Keywords:** metallo-polymer, polymersome, self-assembly, terpyridine

## Abstract

Polystyrene-*b*-polyethylene glycol (PS-*b*-PEG) amphiphilic block copolymers featuring a terminal tridentate *N,N,N*-ligand (terpyridine) were synthesized for the first time through an efficient route. In this approach, telechelic chain-end modified polystyrenes were produced via reversible addition-fragmentation chain-transfer (RAFT) polymerization by using terpyridine trithiocarbonate as the chain-transfer agent, after which the hydrophilic polyethylene glycol (PEG) block was incorporated into the hydrophobic polystyrene (PS) block in high yields via a thiol-ene process. Following metal-coordination with Mn^2+^, Fe^2+^, Ni^2+^, and Zn^2+^, the resulting metallo-polymers were self-assembled into spherical, vesicular nanostructures, as characterized by dynamic light scattering and transmission electron microscopy (TEM) imaging.

## 1. Introduction

**Figure 1:**
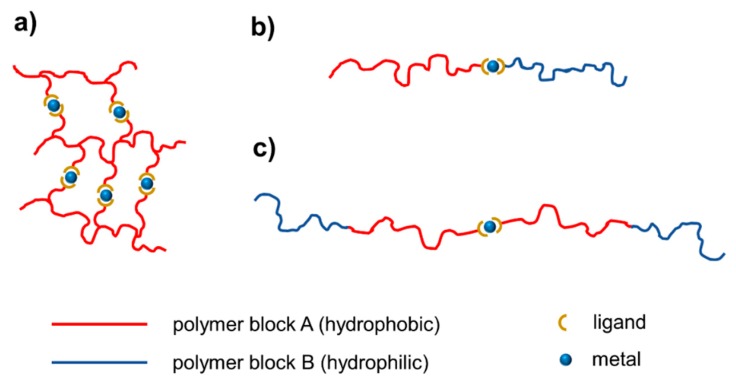
Schematic structures of metallo-polymeric materials: (**a**) cross-linked type, (**b**) amphiphilic block copolymer of the type A-metal-B, and (**c**) amphiphilic block copolymer of the type BA-metal-AB.

Metal-containing polymer assemblies have demonstrated potential as smart soft materials in optoelectronics and biomedical applications, and they have recently attracted attention in the area of self-healing materials due to the dynamic bond formation between ligand-decorated polymeric chains and the appropriate metal ions [1,2,3,4]. For example, a number of Zn-containing metallo-polymers can form gels that self-repair upon photo-induced phase transition that is driven by the dissociation of the metal-ligand bonds [5,6,7,8]. In another example, the addition of Zn^2+^ ions to interlocked polymers leads to increased rigidity of the resulting metallo-polymer [9]. Likewise, mechanically damaged polymeric networks that incorporate polyimine-Cu motifs exhibit self-healing properties when irradiated with UV light as a result of the Cu-N binding [10]. In addition to light stimuli, pH-responsive metallo-polymers have been designed. For example, particles self-assembled from Cu^2+^ metallo-polymers have been demonstrated to respond to changes in pH [11]. Another example is inspired by marine organisms and, as a result of the pH-sensitivity of Fe(III)-catecholate complexes [12] that sacrificially disassemble upon ligand protonation, can transition to a reduced elastic modulus leading to polymer-state liquefaction [13,14]. In the area of drug delivery, Ru-containing metallo-polymers have demonstrated a remarkable capability in delivering and efficiently releasing a Ru-based anticancer drug upon near-IR irradiation [15,16]. In such examples of stimuli-responsive metallo-polymer materials, ligands have been introduced either as side-chains of the polymeric backbone that lead to metallo-gels [17] (Figure 1a) or as end-chains of either the hydrophobic and hydrophilic blocks that lead to the formation of amphiphilic copolymers that are linked together by metal coordination (Figure 1b) [18,19,20,21]. However, to our knowledge, efficient synthetic strategies toward end-chain decorated amphiphilic copolymers that are capable of forming nanovesicles upon metal coordination (Figure 1c) have not been reported and are the focus of this work.

Amphiphilic block copolymers are a class of modular polymers with versatile self-assembly behavior [22,23]. Depending on the chemical nature, length, and self-assembly process, such polymers may generate macromolecular morphologies, such as solid spheres [24], worms [25], and vesicles [26]. In particular, polymeric vesicles [27,28,29] have received increased attention due to their application as transport and delivery platforms in biomedicine [30,31,32,33] and also as nano-containers in complex catalytic systems [34,35,36]. Here, we introduce a versatile synthetic strategy that allows for the efficient preparation of terminally ligand-decorated amphiphilic block copolymers and access to their corresponding metallo-polymer assemblies. Such amphiphilic systems can potentially introduce new functional properties, owing to the metal coordination at the hydrophobic, internal interface of two amphiphilic chains. We tested this strategy through the synthesis, characterization, and self-assembly of terpyridine-appended polystyrene-*b*-polyethylene glycol (PS-*b*-PEG) block copolymers and derived metal complexes integrating all of the components and their associated properties into the resulting coordination macromolecules (Figure 1c).

## 2. Materials and Methods

### 2.1. General Materials and Techniques

All of the solvents (ACS reagent grade, ≥99.5%), chemicals (Sigma-Aldrich, St. Louis, MO, USA) and Amberlite anion-exchange resin (Sigma-Aldrich, 216569) were used as received without further purification, unless stated otherwise. Styrene (Sigma-Aldrich, S4972) was passed through a short alumina column immediately prior to use. The bis(4′-(*p*-methylenephenyl)-2,2′:6′,2″-terpyridine)-trithiocarbonate precursor (**tpy-CTA**) was prepared following a literature procedure [37]. Dialysis was performed using Thermo Scientific Slide-A-Lyzer® 3.5K dialysis cassettes (Thermo Fisher Scientific, Waltham, MA, USA). Nuclear magnetic resonance (NMR) spectra were recorded on a Bruker 500 instrument (Bruker, Billerica, MA, USA) at room temperature, while using CDCl_3_ as solvent. The chemical shifts were calibrated according to the residual protonated CHCl_3_ solvent resonance. Gel permeation chromatography (GPC) was performed on Malvern Viscotek instrument (Malvern Panalytical, Malvern, UK) that was equipped with a T5000 column using pre-filtered 1 mg mL^−1^ polymer solution in inhibitor-free HPLC grade tetrahydrofuran at room temperature. The averaged molecular weight and polydispersity are reported against a calibration curve based on freshly generated polystyrene narrow standards. Transmission electron microscopy (TEM) micrographs were obtained either on a JEOL 1200 (JEOL, Tokyo, Japan), operating at 120 kV, which was equipped with an ORCA-Flash v2.8 CMOS digital camera (Hamamatsu Photonics K. K., Japan) or on a FEI Titan ETEM (Thermo Fisher Scientific, Waltham, MA, USA), operating at 300 kV and equipped with a BM-Ceta camera (Thermo Fisher Scientific). The samples were deposited by pipetting ~10 µL of a 0.1 mg mL^−1^ aqueous solution onto 300 mesh copper grids with a thin carbon film (Cu-300CN, Pacific Grid-Tech, San Francisco, CA, USA). The grids were then placed in a vacuum desiccator overnight before imaging. Because of the low contrast of low-atomic number elements in TEM, the images were taken very slightly out of the focal plane to capture particle morphology. Dynamic light scaterring (DLS) analysis of aqueous polymer and metallo-polymer solutions was performed on a Zetasizer NanoZS 632 nm (Malvern Panalytical, Malvern, UK) using BrandTech 3mL PMMA cuvettes (BrandTech Scientific, Essex, CT, USA). Samples were analyzed at 22.0 °C for ≥ 60 s with automated positioning and attenuation. Within the Zetasizer software (Version 6.34, Malvern Panalytical, Malvern, UK), the dispersant was set to water, with refractive index n = 1.330 and viscosity η = 0.9540 cP. The sample viscosity was taken to be the dispersant viscosity value. Note that, because the amount of water inside the polymer particles is unknown, it is not possible to estimate the average particle refractive index. Therefore, only size distributions that are based on scattering intensity (and not normalized for volume or number) are reported, since additional normalizations require an estimate of refractive index.

### 2.2. Synthesis and Characterization

**tpy-PS_1_:** A 1:1000 mixture of **tpy-CTA** (1.504 g, 1.0 mmol) and styrene (208.5 g, 1.0 mol) was degassed by three freeze-pump-thaw cycles, backfilled with dry argon, and then heated to 110 ^o^C for 12 h. The mixture was then poured slowly into cold methanol and the resulting solid was collected by filtration and then further purified by precipitation of a CH_2_Cl_2_ solution in cold methanol. The collected solid was dried in vacuum at room temperature for 24 h. Monomer conversion: 11% (22.9 g), M*_n_* = 8,800 Da, *Ð* = 1.36. Further confirmation by ^1^H-NMR spectrum is in agreement with previously reported polymers [37].

**tpy-PS_2_ and tpy-PS_3_:** A solution of **tpy-PS_1_** (2.2 g) in styrene (52.1 g) was degassed by three freeze-pump-thaw cycles, backfilled with dry argon, and heated to 110 ^o^C for 6 h and 12 h, respectively, to generate the elongated telechelic polymers **tpy-PS_2_** and **tpy-PS**_3_. Quenching and purification of the polymers are identical to those that are described for **tpy-PS_1_**. Yield: 19% (10.3 g), M*_n_* = 23,500 Da, *Ð* = 1.23 (**tpy-PS_2_**); yield: 25% (13.6 g), M*_n_* = 48,200 Da, *Ð* = 1.16 (**tpy-PS_3_**).

**tpy-PS_1-3_-PEG_43_**: Solutions of **tpy-PS_1_** (2.2 g) in 10 mL of dimethylformamide (solution I), 1.0 M *n*-BuNH_2_-Et_3_N (1:1 ratio) in dimethylformamide (solution II), and acryloyl methoxyether-PEG (mPEG_43_; 6.0g; 3.0 mmol) in 10 mL dimethylformamide (solution III) were each degassed with dry argon for 10 min. 10 mL of solution II was added to solution I, and the resulting mixture was allowed to stir at room temperature for 20 min, followed by the addition of solution III and stirring under argon atmosphere for 4 h. The resulting solution was then added dropwise to 200 mL of ice-cold methanol under slow stirring (200 rpm). The precipitate was collected by filtration and then further purified by precipitation of the tetrahydrofuran copolymer solution into ice-cold methanol. Yield: 6.3 g (80.8%) of **tpy-PS_1_-PEG_43_**. ^1^H-NMR (CDCl_3_, 500MHz, *δ*): 8.70–8.78 (m, 1H, tpy), 7.89–7.96 (m, 0.4H), 7.74–7.82 (m, 0.3H, tpy), 7.36–7.40 (m, 0.5H, tpy), 6.90–7.23 (br, 23H, PS-aromatic), 6.30–6.85 (br, 14H, PS-aromatic), 3.69 (s, 4H, PEG-C*H*_2_-), 1.75–2.20 (br, 8H, PS-C*H*-), 1.35–1.60 (br, 16H, PS-C*H*_2_-); ^13^C-NMR (CDCl_3_, 125MHz, *δ*): 156.39, 155.95, 155.91, 150.27, 149.83, 148.55, 145.73, 145.34, 128.96, 128.64, 128.33, 127.60, 127.43, 126.99, 126.30, 126.13, 125.11, 43.99, 40.97, 40.82, 39.97.

**M(tpy-PS_1-3_-PEG_43_)_2_ (M = Mn^2+^, Fe^2+^, Ni^2+^, Zn^2+^):** The metallo-polymers were generally prepared by overnight mixing the amphiphilic polymers in CH_2_Cl_2_ with triflic salts of the metal ions, M(OTf)_2_, followed by precipitation of the resulting metallo-polymers into cold methanol. For example, **tpy-PS_1_-PEG_43_** (0.116 g, 0.02 mmol) was dissolved in 10 mL of CH_2_Cl_2_ in a 20 mL scintillation vial, to which Zn(OTf)_2_ (4.0 mg, 0.01 mmol) in 100 μL of methanol was added. The M*_n_* obtained by GPC were used for stoichiometric calculations. The mixture was then allowed to stir overnight, followed by concentration of the final solution to *ca*. 1 mL. The concentrate was added dropwise to 20 mL of ice-cold methanol and the resulting solid material was collected by filtration and then dried in vacuum at room temperature for 24 h.

Polymers and metallo-polymers were dissolved in tetrahydrofuran or dimethylformamide to obtain a concentration of 1.0 mg mL^−1^. Deionized water (DIW; 18.2 mΩ) was added to these solutions at 1 mL h^−1^ rate with constant stirring (900 rpm) until the ratio of organic solvent/DIW reached 50%, followed by the dropwise addition of 1 mL of the resulting turbid solution to vigorously stirred DIW (10 mL). The remaining organic solvent was removed by dialyzing the turbid solutions for 24 h against DIW using a 3.5 kDa membrane.

## 3. Results

### 3.1. Synthesis and Charachterization of Polymers and Metallo-Polymers

**Scheme 1:**
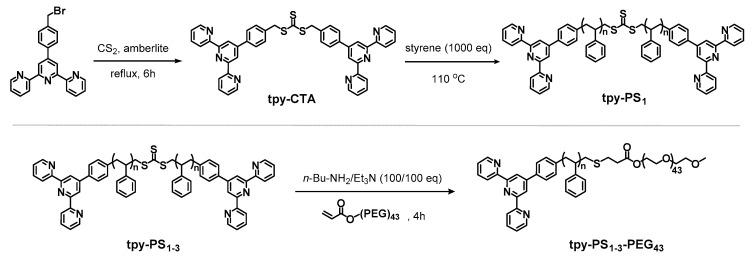
Synthetic scheme for the ligand-functionalized amphiphilic block copolymers tpy-PS_1-3_-PEG_43_.

In our studies, the *N,N,N*-tridentate terpyridine (tpy) is the ligand of choice due to its synthetic versatility [38] and capability to coordinate a variety of metal ions [39]. Terpyridine has been introduced into polystyrene through anionic [40], living-free radical [41], and reversible addition-fragmentation chain-transfer (RAFT) polymerization techniques [42], as well as into PEG by simple ether formation between deprotonated hydroxyl groups and terpyridine aryl halides [17]. However, in cases where the ligand was not part of the polymeric backbone, it was either connected to both chain ends of the polymer or connected to one chain-end of polymer without the possibility of simple chemical modification of the second end (*n*-Bu, *sec*-Bu) [40]. Here, we utilize RAFT polymerization of styrene for the preparation of the ligand-modified hydrophobic polystyrene block due to the chemical versatility of the remaining sulfur-based RAFT chain-end group [43,44], and the symmetric bis-terpyridine-trithiocarbonate was chosen as the chain transfer reagent (**tpy-CTA**, Scheme 1) due to its synthetic simplicity and the ease of generating multi-gram quantities from widely available starting materials in a single step. We have also found that changing the purification method from a frequently used re-crystallization in carbon disulfide to a sequential wash with ethanol/acetone/hexane decreases the purification time and increases the isolated yield of the product from the reported 68% [37] to 89%.

Initially, styrene polymerization was carried out with **tpy-CTA** at 110 ^o^C for 12 h, generating the telechelic **tpy-PS_1_** (8,800 Da) used as a starting material for the block copolymer construction and, alternatively, as a macro **tpy-CTA** for generation of the higher molecular weight polystyrenes **tpy-PS_2_** (23,500 Da) and **tpy-PS_3_** (48,200 Da) (Table 1). The resulting **tpy-PS_1–3_** were then used to prepare the target ligand-modified amphiphiles in good yields of isolated product (80–88%) by one-pot stepwise conjugation of acryloyl methylether-PEG_43_ (mPEG) by chemical modification of the trithiocarbonate group. First, **tpy-PS_1–3_** was cleaved to yield the corresponding thiols in the presence of excess *n*-BuNH_2_/triethylamine mixture, followed by the *thiol-ene* reaction between the formed thiol and the double bond of the acryloyl end-group of mPEG (Scheme 1) to then generate the desired **tpy-PS_1–3_-PEG_43_** amphiphilic block copolymers (Table 1). The analysis of block copolymer structure by ^1^H-NMR (Figure S1a) indicated the expected increase in the intensity of the polystyrene signals due to the change in the molecular weight of the hydrophobic block. Further analysis by GPC corroborated the clean conversion from telechelic **tpy-PS_1–3_** into corresponding **tpy-PS_1–3_-PEG_43_** (Figure S1b). The molecular weights of the resulting block copolymers appear to be lower than the values that were calculated by using the M*_n_*(mPEG) of 1700 Da (Figure S1b). To confirm the M*_n_* of the polymer, UV-Vis titration was performed for **tpy-PS_1_-PEG_43_** with Zn(OTf)_2_ as a standard (Figure 3). The obtained M*_n_* of 5990 Da corroborates the formation of the block copolymer with a molecular weight near the calculated value. It is worth noting that the trithiocarbonate-mediated RAFT polymerization of **tpy-PS_1-3_** can be generalized to produce other amphiphilic block copolymers by growing acrylic acid hydrophilic blocks [45], further broadening the scope of this synthetic methodology that is based on the generation of telechelic polymers.

**Figure 2:**
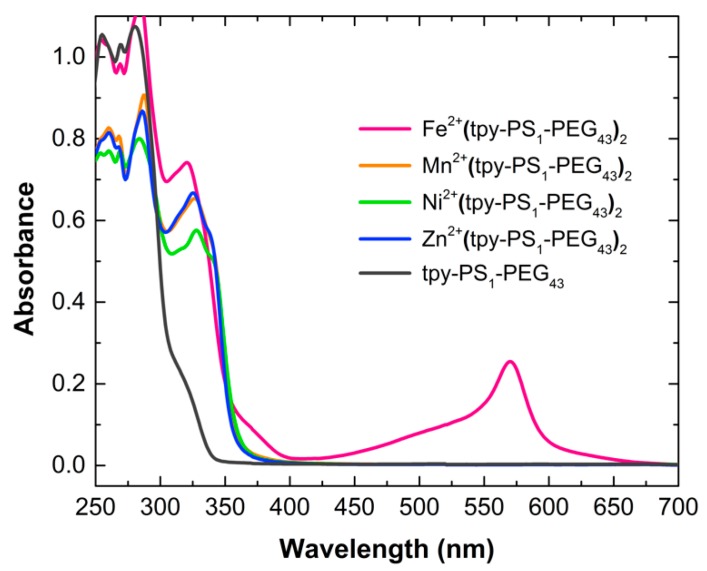
UV-Vis absorption spectra of **tpy-PS_1_-PEG_43_** and its derived metallo-polymers, **M(tpy-PS_1_-PEG_43_)_2_**, in dichloromethane solutions at a typical sample concentration of 0.03 mM.

**Figure 3:**
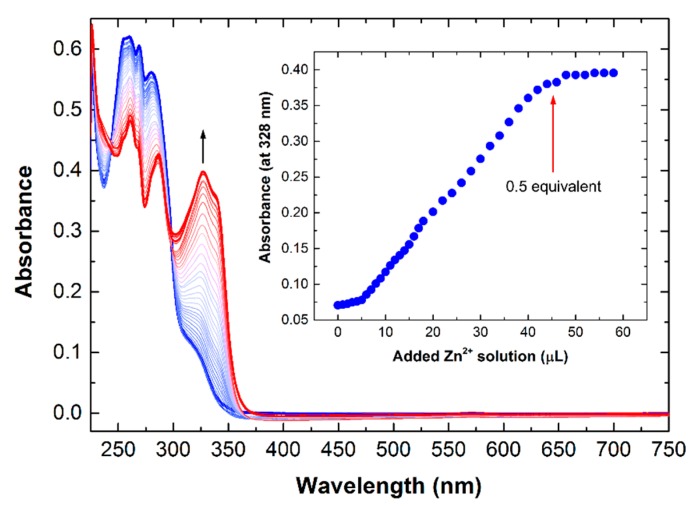
UV-Vis spectrophotometric titration of **tpy-PS_1_-PEG_43_** with a concentrated solution of Zn(OTf)_2_ in dichloromethane. The arrow indicates the number of added equivalents corresponding to the polymer 2:1 metal stoichiometry, in agreement with the **M(tpy-PS_1_-PEG_43_)_2_** formulation.

Polymers **tpy-PS_1_-PEG_43_** were then reacted with triflic metal salts M(OTf)_2_ in a 2:1 ratio to generate the corresponding metallo-polymers **M(tpy-PS_1_-PEG_43_)_2_,** as depicted in Figure 1c (where M = Mn^2+^, Fe^2+^, Ni^2+^, or Zn^2+^). The metal coordination was spectroscopically confirmed by observation of the characteristic UV-Vis absorption bands of the corresponding M(tpy)_2_ motifs [46,47,48,49,50] (Figure 2), which emerged during the reaction and allowed for the quantitative analysis of the degree of metal-ligand binding on the amphiphilic polymers via spectrophotometric titration of the **tpy-PS_1_-PEG_43_** with M(OTf)_2_ (as exemplified for M = Zn^2+^ in Figure 3). Metal coordination involving the diamagnetic species Zn^2+^ was also corroborated by ^1^H-NMR spectroscopy, which clearly showed a shift of the tpy ligand-based signals upon the addition of Zn(OTf)_2_ to the CDCl_3_ solution of the functionalized copolymer **tpy-PS_1_-PEG_43_** (Figure S2).

### 3.2. Self-Assembly of Metallo-Polymers

**Figure 4:**
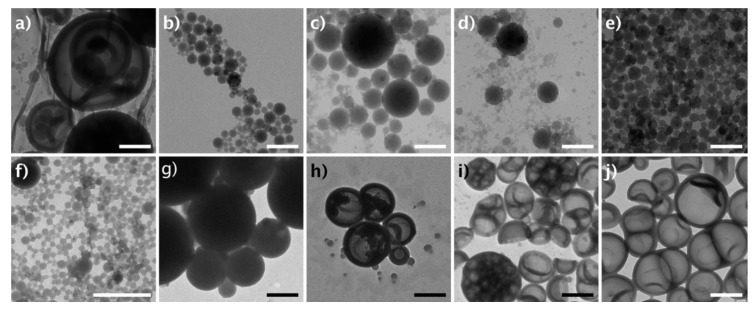
Transmission electron microscopy (TEM) micrographs of dehydrated nanostructures assembled by water infusion of (**a**) **tpy-PS_1_-PEG_43_**, (**b**–**e**) **M**(**tpy-PS_1_-PEG_43_)_2_**, (**f**) **tpy-PS_2_-PEG_43_**, and (**g**–**j**) **M(tpy-PS_2_-PEG_43_)_2_** solutions in tetrahydrofuran [M = Zn^2+^ (**b**,**g**); Ni^2+^ (**c**,**h**); Fe^2+^ (**d**,**i**); Mn^2+^ (**e**,**j**)]. Scale bars represent 500 nm. All of the micrographs have the same dimensions, with the exception of (**f**), where magnification is higher to enable visual inspection of individual particles with sufficient resolution.

With the formation of metallo-polymers shown, we investigated their self-assembly behavior along with that of the parent metal-free polymers. The self-assembly of all **tpy-PS_1-3_-PEG_43_** and **M(tpy-PS_1_-PEG_43_)_2_** species was induced by exchanging the water-miscible organic solvent (tetrahydrofuran or dimethylformamide) with deionized water (DIW) by the dropwise addition of DIW into stirred solutions of the polymeric material in organic solvent. We found that both the metal-free and metal-coordinated species assembled into spherical nanostructures of similar sizes between 170–900 nm, with varying degrees of particle polydispersity (Figure 4, Figure S3 and Table S1). The size and dispersity of the samples were characterized by dynamic light scattering (DLS) and correlated by particle size analysis via transmission electron microscopy (TEM) (Figure S4).

Owing to our specifically targeted design of amphiphilic polymers, both the **tpy-PS_1_-PEG_43_** and **M(tpy-PS_1_-PEG_43_)_2_** series fit well into the *f*_(PS)_ > *f*_(PEG)_ category (where *f* is volume fraction of the corresponding polymer), which is expected to lead to the formation of mixtures of multi- and unilamellar spherical polymersomes [51] (Figure 4 and Figure S3). Indeed, TEM micrographs of several samples indicate the formation of polymersomes (Figure 4a,h,i,j), in agreement that hollow polystyrene-based polymersomes adopt a concaved shape during exposure to constant withdrawal of organic solvent from the hydrophobic inner layer during dialysis [52,53], as well as drying of the polymersome solution [54]. Other particles that appear to be filled (e.g., Figure 4g) are similar to structures that were previously assigned to large compound micelles [55].

Interestingly, a comparison across both metal-free and metal-coordinated series suggests that the size and shape of these polymersomes are not determined exclusively by the molecular weight of the PS block (Table S1). We have found that increasing the molecular weight of the hydrophobic block within the amphiphilic polymers leads to aggregation and precipitation of the material, generating lower yielding solutions of polymersomes. Structural effects that are potentially induced by the metal-ligand moieties will therefore be systematically explored in subsequent studies.

## 4. Conclusions

A high-yielding and versatile synthetic strategy toward ligand-terminated amphiphilic block copolymers was successfully developed and demonstrated in this work. Their corresponding metallo-polymers can self-assemble into metal-coordinated polymersomes. Polymer block sizes and metal coordination influence the morphology of self-assembled nanostructures, although a detailed understanding of the exact roles of each will require further investigation. While the introduction of metal-ligand motifs can be exploited to potentially impart new optical, electronic, and magnetic properties into the design of stimuli-responsive metallo-polymer assemblies, the structural tunability of target (nano)materials, such as polymersomes, must also be considered. Further work seeks to understand the nature of metal/ligand-induced structural effects, as well as to fully explore functional, stimuli-responsive activities in these metallo-polymer vesicles.

## Figures and Tables

**Table 1:** Molecular weight (M*_n_*) and dispersity (*Ð*) of the telechelic **tpy-PS_1-3_** and amphiphiles **tpy-PS_1-3_-PEG_43_**, as characterized by gel permeation chromatography (GPC).

Name	M*_n_*	*Ð*	Name	M*_n_*_(calc)_	M*_n_*_(GPC)_	*Ð*
**tpy-PS_1_**	8800	1.36	**tpy-PS_1_-PEG_43_**	6078	5800	1.38
**tpy-PS_2_**	23,500	1.23	**tpy-PS_2_-PEG_43_**	13,428	11,200	1.36
**tpy-PS_3_**	48,200	1.16	**tpy-PS_3_-PEG_43_**	25,778	23,300	1.28

## Supplementary Materials

The following are available online at http://www.mdpi.com/1996-1944/12/4/601/s1. Figure S1: characterization of telechelic and amphiphilic polymers; Figure S2: characterization of Zn^2+^(tpy-PS_1_-PEG_43_)_2_ by ^1^H-NMR spectroscopy; Figure S3: TEM micrographs of nanostructures assembled using water infusion into a solution of Mn^2+^(tpy-PS_1-3_-PEG_43_)_2_ in tetrahydrofuran and dimethylformamide for varying polystyrene molecular weights; Figure S4: diameter of particles formed by self-assembly of various metallo-polymer species dissolved in varying organic solvents prior to solution exchange into water; Table S1: DLS size analysis and dispersity of the metallo-polymersomes.
