# Supplementary material for: Synthesis of Terpyridine-Terminated Amphiphilic Block Copolymers and Their Self-Assembly into Metallo-Polymer Nanovesicles
Source: Materials (Basel). 2019 Feb 17;12(4):601. doi: 10.3390/ma12040601 (PMC6416640; doi:10.3390/ma12040601)
Supplement: Supplementary file 1 [file materials-12-00601-s001.pdf]

Synthesis of Terpyridine-Terminated Amphiphilic Block Copolymers and their Self-Assembly into Metallo-Polymer Nanovesicles

Tatyana Elkin [1], Stacy M. Copp [1], Ryan L. Hamblin [1], Jennifer S. Martinez [1,2], Gabriel A. Montaño [1,2,*] and Reginaldo C. Rocha [1,*]

1 Los Alamos National Laboratory, Center for Integrated Nanotechnologies, Los Alamos, NM 87545, USA; tatyana_elkin@lanl.gov, smcopp@lanl.gov, rhamblin@lanl.gov, rcrocha@lanl.gov
2 Northern Arizona University, Department of Chemistry and Biochemistry, Flagstaff, AZ 86011, USA; jennifer.martinez@nau.edu, gabriel.montano@nau.edu
* Correspondence: rcrocha@lanl.gov; Tel.: +1-505-665-0124, gabriel.montano@nau.edu; Tel.: +1-928-523-2353

Supplementary Materials

Characterization of the amphiphilic block co-polymers

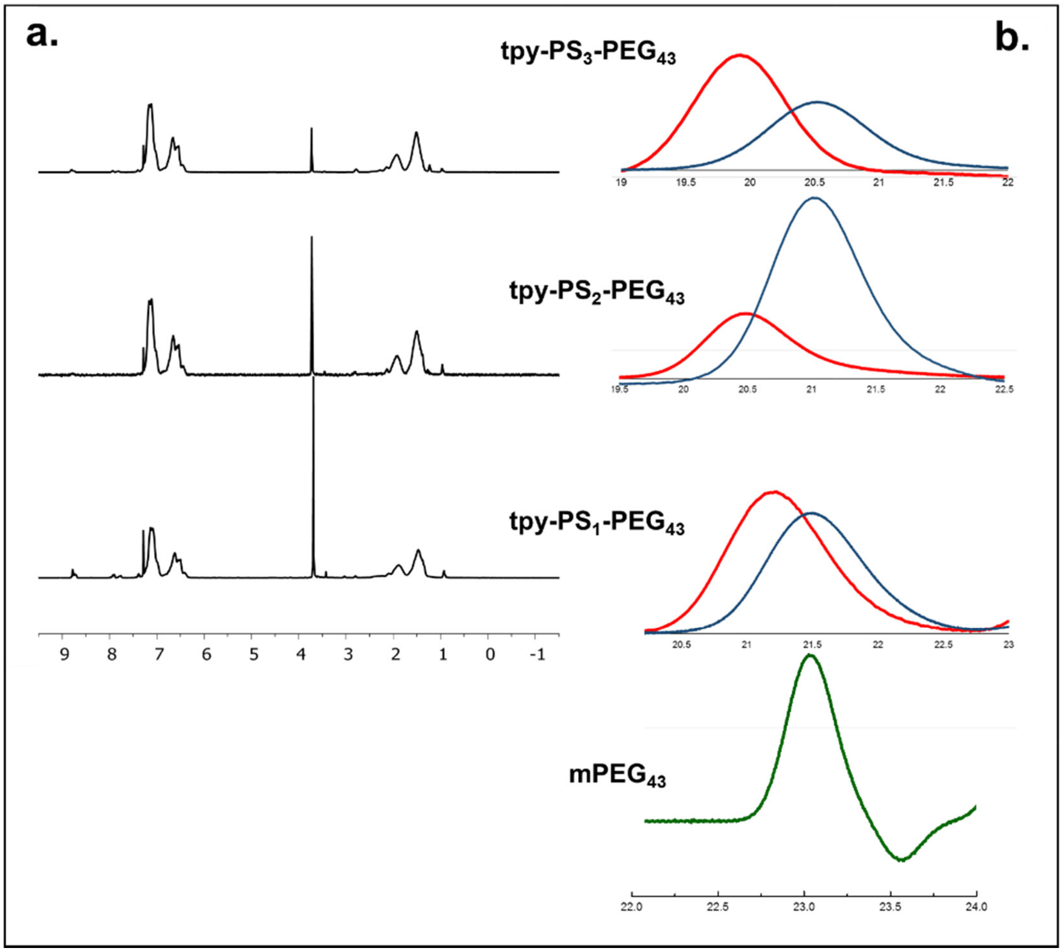

Figure S1. Characterization of telechelic and amphiphilic polymers: a) ^{1}H-NMR spectra of amphiphilic block copolymers demonstrating the difference in intensity of the signals for PEG vs PS. b) GPC traces of amphiphilic block copolymers (blue) vs corresponding telechelic parent polystyrenes (red) as well as mPEG (green) for comparison.

Characterization of Zn^{2+}(tpy-PS$_1$-PEG$_{43}$)$_2$ by ^{1}H-NMR spectroscopy

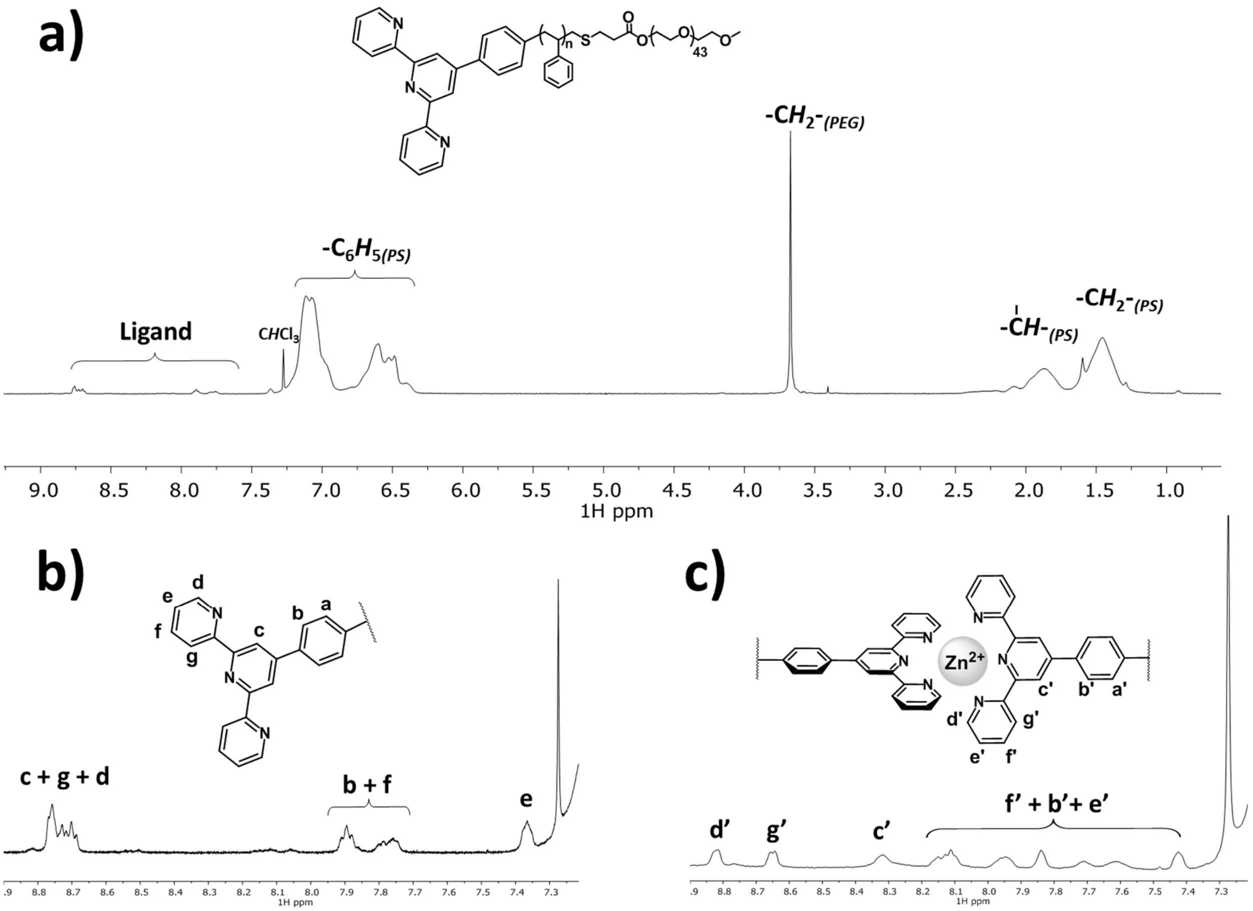

Figure S2. **a)** Full ^{1}H-NMR spectrum of the amphiphilic block copolymer **tpy-PS$_1$-PEG$_{43}$**; **b)** ^{1}H-NMR spectrum of the amphiphilic block copolymer **tpy-PS$_1$-PEG$_{43}$** showing the region of the metal-free terpyridine ligand **(**signal **e** is overlapping with the C_6H_5 signals of the PS block**),** in agreement with previously reported assignment [1]; **c)** ^{1}H-NMR spectrum of the metallo-polymer **Zn^{2+}(tpy-PS$_1$-PEG$_{43}$)$_2$** showing the region of the metal-coordinated terpyridine ligand **(**signal **a'** is overlapping with the C_6H_5 signals of the PS block**)**, in agreement with previously reported assignment for similar zinc(II) bis-terpyridine systems [2].

1. Zhang, L.; Zhang, Y.; Chen, Y. *Eur. Polym. J.* **2006**, *42*, 2398-2406, DOI:10.1016/j.eurpolymj.2006.05.017.
2. Tessore, F.; Ugo, D. R.; Pizzotti, M. *Inorg. Chem.*, **2005**, *44*, 8967–8978, DOI: 10.1021/ic050975q.

Self-assembly of M^{2+}(tpy-PS$_{1\text{-}3}$-PEG$_{43}$)$_2$ metallo-polymers ($M = Mn^{2+}$, Fe^{2+}, Ni^{2+}, Zn^{2+})

Table S1. DLS analyzed size and dispersity of the metallo-polymersomes **M(tpy-PS$_{1\text{-}3}$-PEG$_{43}$)$_2$** formed by H_2O/DMF exchange.

M	tpy-PS$_1$-PEG$_{43}$		tpy-PS$_2$-PEG$_{43}$		tpy-PS$_3$-PEG$_{43}$	
	d [nm]	*Đ*	d [nm]	*Đ*	d [nm]	*Đ*
none	353.8±102.5	0.356	581.4±166.0	0.080	832.7±429.4	0.184
Zn^{2+}	436.7±120.2	0.078	n.a.*	0.493	477.8±160.4	0.201
Ni^{2+}	234.6±86.72	0.182	380.6±143.9	0.170	169.8±79.52	0.193
Fe^{2+}	323.9±139.5	0.235	1373.0±407	0.315	376.8±199.3	0.541
Mn^{2+}	422.1±210.5	0.195	n.a.*	0.409	909.3±246.4	0.434

* Due to poor fitting to the correlation curve, the values for the size of the particles (276.1±38.47 nm for Zn^{2+} and 345.7±57.29 nm for Mn^{2+}) are not included in the Table.

We found that metallo-polymers self-assemble into spherical nanostructures upon exchange from organic solvent to DIW, regardless of the organic solvent used to dissolve the metallo-polymers or the molecular weight of the PS block. Figure S3 shows spherical particle morphologies with varying solvents and PS molecular weights for the metallo-polymer with $M = Mn^{2+}$.

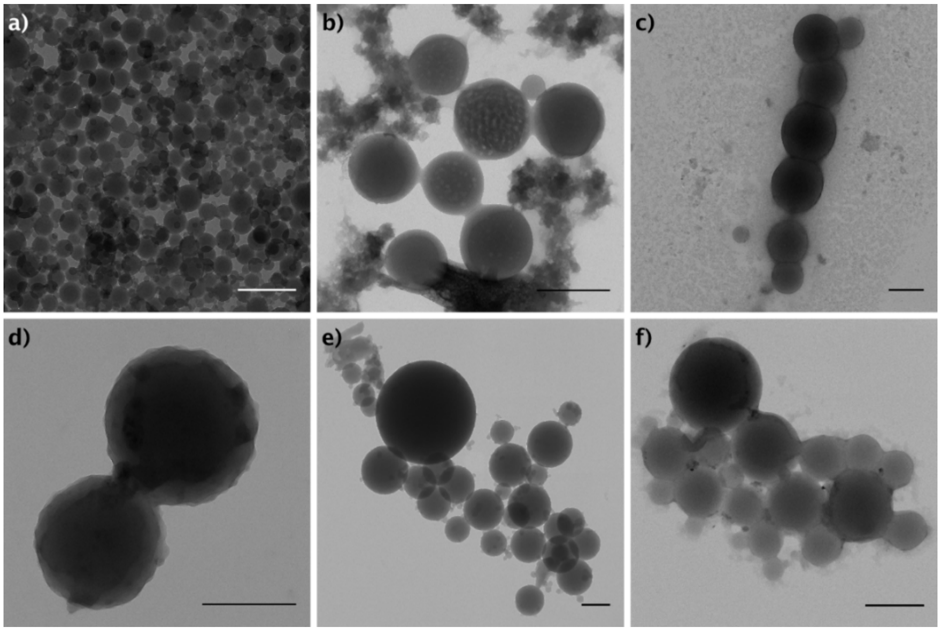

Figure S3. TEM micrographs of nanostructures assembled using water infusion into a solution of $\mathbf{Mn^{2+}(tpy\text{-}PS_{1\text{-}3}\text{-}PEG_{43})_2}$ in THF (**a-c**) and DMF (**d-f**) for various polystyrene molecular weights: PS_1 (**a,d**), PS_2 (**b,e**), and PS_3 (**c,f**). Scale bars represent 500 nm.

Particle analysis of TEM images was performed using the ImageJ software package. Metallo-polymer nanoparticles were outlined with the oval selection tool and measured for Feret's diameter, with the median value taken as the particle diameter. Resulting measurements were binned and plotted for comparison with DLS data using the Igor Pro software package. Figure S3 compares particle diameter determined from DLS intensity distributions (black lines, left axes) and from size analysis of TEM micrographs (blue bars, right axes). DLS-determined diameter is expected to be larger than TEM-measured diameter because scattering intensity scales with particle radius to the sixth power, weighting intensity distributions to larger particle diameters. Thus, particle diameter is routinely overestimated by DLS-measured intensity distributions, and this is particularly significant when a small population of large particles coexists with a large population of small particles. For example, an exponentially decreasing size distribution proportional to e^{-d} (diameter d) would have a DLS intensity distribution proportional to $e^{-d} * d^6$, appearing somewhat Gaussian in nature. This is indeed the case in Figures S3 b) and e). Such scaling must be taken into account when comparing the size distributions measured by DLS and by TEM. Note that transformation of DLS intensity distribution to either volume or number distribution requires precise knowledge of particle refractive index (see experimental section). Because the degree to which particles are hollow or filled is not precisely known and/or may vary, we do not perform this transformation and instead report only intensity distributions. Figure S3 thus shows that size determination by TEM and DLS agree to within the expected overestimation of diameter by DLS, suggesting that TEM micrographs of dehydrated metallo-polymer nanoparticles formed by self-assembly are qualitatively representative of particle size in solution.

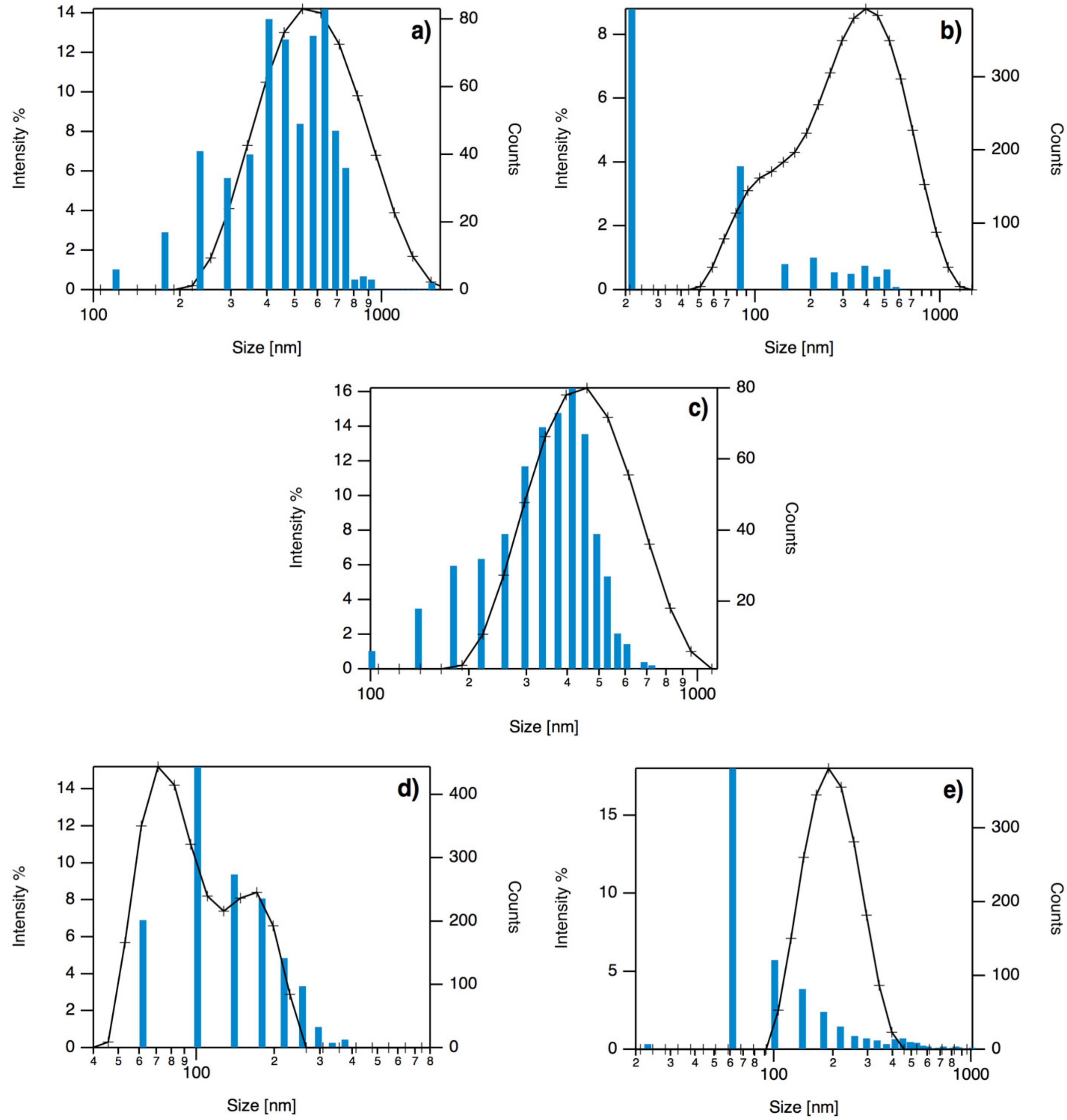

Figure S4. Diameter of particles formed by self-assembly of various metallo-polymer species dissolved in varying organic solvents prior to solution exchange into DIW. Black lines, left axes: DLS intensity distribution. Blue bars, right axes: histogram counts of diameters measured from TEM micrographs. (a) **Fe^{2+}(tpy-PS$_1$-PEG$_{43}$)$_2$** in DMF, (b) **Fe^{2+}(tpy-PS$_1$-PEG$_{43}$)$_2$** in THF, (c) **Fe^{2+}(tpy-PS$_1$-PEG$_{43}$)$_2$** NMP, (d) **Fe^{2+}(tpy-PS$_3$-PEG$_{43}$)$_2$** in DMF, and (e) **Zn^{2+}(tpy-PS$_3$-PEG$_{43}$)$_2$** in DMF.
